# Assessing the impact of dietary choices on fiber deficiency: insights from the 2017–2020 Polish national adult nutrition survey

**DOI:** 10.3389/fnut.2024.1433406

**Published:** 2024-09-13

**Authors:** Alicja Kucharska, Beata Irena Sińska, Mariusz Panczyk, Piotr Samel-Kowalik, Dorota Szostak-Węgierek, Filip Raciborski, Bolesław Samoliński, Iwona Traczyk

**Affiliations:** ^1^Department of Human Nutrition, Faculty of Health Sciences, Medical University of Warsaw, Warsaw, Poland; ^2^Department of Education and Research in Health Sciences, Medical University of Warsaw, Warsaw, Poland; ^3^Department of Prevention of Environmental Hazards, Allergology and Immunology, Faculty of Health Sciences, Medical University of Warsaw, Warsaw, Poland; ^4^Department of Clinical Dietetics, Faculty of Health Sciences, Medical University of Warsaw, Warsaw, Poland; ^5^Department of Public Health, Faculty of Health Sciences, Medical University of Warsaw, Warsaw, Poland

**Keywords:** dietary fiber intake, high-fiber food products, guideline adherence, healthy diet, Polish population

## Abstract

**Introduction:**

Dietary fiber is a key component of a healthy diet, associated with a reduced risk of cardiovascular disease, obesity, type 2 diabetes, certain cancers, chronic inflammation, or depression. The aim of the study was to perform an in-depth analysis of dietary fiber intake in the Polish population, taking account of the consumption of groups of products that are fiber sources and identify any age-related differences in the dietary fiber intake of the subjects.

**Methods:**

We analyzed data obtained from two representative cross-sectional studies on the diet and nutritional status of adult Polish residents including the total of 4,000 individuals aged 19 years and more. Two 24-h recalls were used per individual to assess the diet using the computer-assisted personal interview (CAPI) technique. Total fiber content and fiber contained in cereal products, vegetables, fruits, legumes, nuts and seeds were calculated. Fiber intake was compared to the recommendations: 25 g/d for adults up to 65 years of age and 20 g/d for those aged 66 years and older. All statistical analyses, including the Pearson’s chi-squared test, the Student’s *t*-test, and the Analysis of Variance (ANOVA), were conducted using STATISTICA^™^ version 13.3, with the results being adjusted for demographic distribution biases to enhance the representativeness.

**Results:**

The average daily fiber intake was 17.83 ± 0.14 g/day (78% of the recommended intake), with 20.5% of respondents meeting the requirement. More men than women (27.05% vs. 14.3%;) met the requirement and men were characterized by a higher average intake (19.34 ± 0.20 g/day) than women (16.43 ± 0.19 g/day). The main fiber sources were cereals (44.1%), vegetables (23.6%), and fruits (16.0%). As regards men, the sources included refined bread (25.8%), vegetables (23.1%), and fruits (10.2%) and for women, they were vegetables (24.0%), fruits (17.2%), and refined bread (16.3%). Although refined bread is not recommended as a primary fiber source due to its lower fiber content compared to whole grain bread, its high consumption significantly contributed to the total fiber intake.

**Conclusion:**

The prevalence of widespread dietary fiber deficiency calls for the intensification of educational efforts that address the health advantages and sources of dietary fiber, as well as methods for its inclusion in daily meals.

## 1 Introduction

Dietary fiber is considered to be a key component of a healthy diet. Ample evidence supported its important role as a contributor to overall metabolic health by influencing glucose and lipid regulation and insulin sensitivity. A higher fiber intake was associated with a reduced risk of cardiovascular disease (1), obesity, type 2 diabetes (2), and certain cancers (3). The health benefits of fiber intake also include the proper functioning of the digestive system and the support of healthy intestinal microbiome, which is a mediator of appetite regulation, chronic inflammation and a lower risk of depression (3). According to research, a potential link was suggested between low fiber intake and polycystic ovary syndrome (PCOS) and irritable bowel syndrome (IBS). Also, increasing fiber intake was recommended as beneficial due to its role in improving gut health and hormonal balance (4, 5).

The term “dietary fiber” encompasses a variety of chemicals characterized by different properties and different effects on the body. The European Food Safety Authority (EFSA) listed non-starch polysaccharides, cellulose, pectins, hydrocolloids, fructooligosaccharides and “resistant starch” (6). Dietary fiber may also be classified based on its solubility in water. The main sources of soluble fiber include fruits and vegetables, while cereals and whole grain food are sources of insoluble fiber, with the majority of naturally available fiber-rich foods containing variable amounts of both soluble and insoluble fiber (7).

Numerous countries and international organizations have established recommendations for fiber content in the diet, typically at 20–38 g/day for adults. The European Food Safety Authority and the World Health Organization recommended 25 g as the sufficient fiber consumption in adults (8, 9). Health guidelines in the USA recommended the fiber intake of 14 g/1000 kcal (25 g/day for women and 38 g/day for men) (10). In Germany, adults are recommended to consume over 30 g/day of fiber (11). According to the Polish guidelines, the diet of adults should provide 25 g/day, and seniors from 66 years of age – 20 g/day (12). Fiber intake exceeding the values may be beneficial for health.

Given the wide availability of fiber in plant products, meeting the established recommendations should not be difficult, especially since the numerical recommendations are supported by product nutritional recommendations most often presented in the form of a plate or pyramid and supplemented with simple messages (13).

According to numerous recommendations (8, 14–17) presenting the correct structure of the whole-day diet, dietary fiber occurs in all its categories. Vegetables and cereal products should constitute the largest part of the diet. Legumes are recommended as products providing protein, and nuts and seeds as sources of healthy fat. These products are also a source of fiber. The consumption of vegetables and whole grains several times every day and fruit once a day should ensure the adequate supply of fiber in the diet. Meeting this requirement could contribute to reducing the risk of developing numerous diet-related diseases, including obesity, cardiovascular diseases, and colon cancer. It might also improve the quality of life of many people struggling with gastrointestinal problems (18, 19).

Although these facts have been known for years, fiber intake is usually insufficient. In the United States, the supply of fiber in the diet of adults is about 16 g/day (20). A comprehensive review of dietary fiber intake in European countries showed slight differences in dietary fiber intake between countries. Fiber intake was 18–24 g/day in men and 16–20 g/day in women, and was similar between age groups. Cereal products (including bread) constituted the largest source of dietary fiber (21). According to data from the 2013–2014 WOBASZ II study (Multicenter Population Health Research) conducted in Poland, the average intake of dietary fiber was 17.5 g/day in women and 20.9 g/day in men (22).

Although previous studies on dietary fiber intake in Poland provided valuable information, they were mainly based on single 24-h recalls or household budget surveys and older nutrient databases. Furthermore, to the knowledge of the authors, any other representative study conducted among Polish residents involved no detailed analysis of the sources of dietary fiber and the differences in their consumption depending on sex and age.

Therefore, having the latest results of dietary studies representative for the Polish adult population at our disposal, and bearing in mind the importance of fiber for the health, we decided to analyze its consumption depending on the sex and age of the respondents in the context of national dietary recommendations. Our aim was to perform an in-depth analysis of dietary fiber intake in the Polish population, taking account of the consumption of product groups that are fiber sources as well as attempts to profile the relationship between dietary choices and fiber intake in relation to the age of the subjects.

Therefore, using the most recent dietary study data representative of the adult Polish population, incorporating repeated 24-h dietary recalls, and the latest nutrient database, we aimed to analyze fiber intake by sex and age against national dietary recommendations. Our objective was to provide an in-depth analysis of dietary fiber intake in the Polish population, considering product groups as fiber sources and profiling the relationship between dietary choices and fiber intake by age.

## 2 Materials and methods

### 2.1 Design

We analyzed data obtained from two representative cross-sectional studies on the diet and nutritional status of 4,000 adult Polish residents. They were conducted between 2017 and 2020. The study, approved by the Institutional Ethical Review Board at the Medical University of Warsaw (Approval Nos. AKBE/163/17 and AKBE/164/17), adhered to the General Data Protection Act (23) and the Declaration of Helsinki (24).

Respondent selection followed the EFSA guidelines (25) and involved probability sampling from a household address database. To enhance the representativeness and reduce costs, stratified and cluster sampling were used. Initially, 500 statistical areas (clusters) were randomly selected from 34,633 units, with selection probability proportional to the number of residents. Residential buildings within each cluster were then chosen using the TERYT-NOBC register and other sources. Eight buildings per cluster (four for each group) were selected, with reserves in case of refusals. One respondent per building was chosen.

Researchers employed the Computer-Assisted Personal Interview (CAPI) technique for 90% of the interviews, switching to the Computer-Assisted Telephone Interview (CATI) technique for 10% during the COVID-19 pandemic. The detailed methodology of those studies was presented in a separate publication (26).

A fundamental component of the analytical process entailed the utilization of weights that reflected the real distribution of attributes including the sex, age, place of residence, and educational level within the Polish population. Those weighting factors were adjusted in accordance with the information derived from the most recent National Population and Housing Census 2021, carried out by the Statistics Poland (27), to guarantee the representativeness of the sample.

### 2.2 Data collection and instruments

A 24-h dietary recall was used for the dietary assessment. The dietary interview was conducted twice by well-trained, experienced interviewers, with an interval of at least 5 days. The interviewers were extensively trained in several areas: understanding portion sizes of packaged products, their availability on the market, preparation methods, recipes and household measures. They were also trained in how to keep the respondents engaged and ensure effective collaboration to obtain accurate dietary data. To account for the variability of eating habits during the week, the study was conducted on different non-consecutive days, including weekdays and weekends. Data were collected evenly across all seasons to consider the seasonality of consumption. Data were continuously checked and validated by the research team nutritionists.

The 24-h dietary recall interviews were conducted through structured stages and questions:

- Recall of all consumed products and dishes: “Can you list everything you ate and drank yesterday from the moment you woke up until you went to bed? Were there any snacks or drinks between your main meals?”
- Description of the type and composition of consumed products, dishes, and beverages: “Can you describe each food and beverage? What ingredients were used? How was the food prepared (e.g., boiled, fried, baked)?”
- Assessment of portion sizes: “Can you estimate the amount of each food or drink you consumed using household measures?”
- Use of specific household items: “Did you use any specific household items like cups, spoons, or plates? Can you describe their size?”
- Logical and nutritional verification of the obtained information: “Is there anything else you remember eating or drinking? Are there any additional details you might have missed?”

Portion sizes were estimated using a photo album of products and dishes.

Interviews were conducted using the CAPI method with the Dieta 5.0 software recommended for scientific studies in Poland, developed by the Institute of Food and Nutrition in Warsaw (28). The software uses Polish data on the nutritional value of products and foods and national nutritional standards. The portion sizes of food and dishes were quantified using household measures (e.g., glasses, cups, spoons), food portions (based on information from food manufacturers), and an album of portion sizes of products and dishes (29). Information on physical activity, smoking, alcohol consumption and socio-demographic data was also collected.

Using the Dieta 5.0 software, the energy value of the studied diets and the daily supply of total carbohydrates and dietary fiber were calculated. Total fiber intake was compared to the reference range of sufficient intake for the Polish population, i.e., 25 g/d for adults up to 65 years of age and 20 g/d for people 66 years of age and older. The age grouping in our study was done according to the guidelines of the Polish Nutritional Standards (12).

The structure of dietary fiber intake was determined by calculating the intake of groups of products being its source. The following groups of recommended sources listed in the current Polish recommendations for healthy nutrition were included (17): cereal products, vegetables, fruits, legume seeds, nuts and seeds. Subsequently, the amount of fiber contained in those product groups was calculated. The share of each product group in the total fiber intake was calculated by dividing the fiber intake of each product group by the total fiber intake and then multiplying by 100. The analysis comprised fiber intake from individual product groups, both products suitable for direct consumption as well as products included in dishes, and composite products. When calculating the amount of consumption of products in order to combine them into appropriate groups, assumptions were made in accordance with the assumptions of the Dieta 5.0 software (28). Details of the product groups analyzed and the conversion coefficients used are shown in Table 1.

**Table 1:** Product groups and conversion coefficients used in the analysis.

Category	Products
Cereal (total cereals)	Flour, pasta, groats, rice, breakfast cereals, bread (total), cereal snacks Flour from cakes, wafers, cookies and biscuits was added to the flour group, and the intake was estimated on the basis of the guidelines for the recipe composition in g per 100 g of the ready-to-eat product.
Refined bread	light rye bread, light rye and wheat bread, wheat bread and rolls
Whole grain bread	whole grain rye bread, whole grain rye and wheat bread, whole grain wheat bread
Vegetables	Vegetables whose consumption was estimated in the edible parts were converted into a marketed product using information on the quantity of waste. The following conversion coefficients were applied: 90 g of frozen vegetables = 100 g of fresh vegetables (marketed product) 80 g of canned vegetables = 100 g of fresh vegetables (marketed product) 20 g of tomato concentrate = 100 g of vegetables rich in carotene 100 g of ketchup = 100 g of vegetables rich in carotene consumption of vegetable juices was not taken into account
Fruits	Fruits whose consumption was estimated in the edible parts were converted into a marketed product using information on the quantity of waste. The following conversion coefficients were applied: 90 g of frozen fruits = 100 g of fresh fruits (marketed product) 80 g of canned fruits = 100 g of fresh fruits (marketed product) 20 g of dried fruits = 100 g of fresh fruits (marketed product) the amount of fruit from jams was added to the total fruit consumption based on the composition consumption of fruit juices was not taken into account
Legumes	Dry legume seeds (marketed product)
Nuts and seeds	Nuts and seeds (marketed product)

The Dieta 5.0 software combines some cereal products into broad categories (e.g., the rice category includes white and brown rice, the pasta category includes refined and whole grain pasta, the groats category includes all types of groats), making it impossible to accurately calculate the intake of whole grain and refined cereals. Therefore, we calculated the total intake of cereal products in the analysis. A full distinction between whole grain and refined products was only possible for bread.

Dietary supplements are not included in the total fiber intake.

When characterizing dietary fiber intake, carbohydrate to fiber ratio was used, assuming the optimal ratio of ≤10: 1 (30).

### 2.3 Statistical analysis

All statistical calculations were conducted within the framework of weighted analysis, ensuring the findings were representative of a broader population structure. This adjustment accounts for potential biases related to demographic distribution and enhances the generalizability of the results. As regards the statistical evaluation of the study, inferential analyses were conducted under the framework of null hypothesis testing. The initial examination of the population’s demographic attributes, comprising the age, residential area, educational attainment, financial situation, and lifestyle factors like physical activity and tobacco use, employed the Pearson’s chi-squared test to discern sex-based distinctions. Central to the analysis was the assessment of dietary intake with a specific focus on average calorie consumption, the distribution of energy across various macronutrients, and fiber intake levels. Descriptive statistical measures, including the means and the standard errors of the mean (SEM), were used to depict the central tendency and dispersion of the data. The comparative analysis of dietary patterns between sexes was performed using the Student’s t-test, facilitating the identification of significant dietary differences. Further categorical data exploration, particularly the proportion of individuals adhering to recommended dietary fiber intake, was accomplished through the chi-squared test for independence. A detailed examination of fiber consumption patterns was conducted using the Analysis of Variance (ANOVA). Such an approach allowed for the comparison of average fiber intake across different food groups and age groups within each sex, encompassing a variety of food sources such as cereals, vegetables, fruits, legumes, and nuts and seeds. The entirety of statistical analyses was executed using STATISTICA^™^ version 13.3 (TIBCO^®^ Software Inc., Palo Alto, California, United States), with a predetermined alpha level of 0.05 for the rejection of the null hypothesis across all statistical tests.

## 3 Results

The characteristics of the studied population by sex are presented in Table 2. The respondents, depending on the sex, differed significantly in terms of sociodemographic characteristics (the place of residence, level of education, current financial situation), and lifestyle elements (physical activity, past and current smoking, current alcohol consumption).

**Table 2:** Characteristics of the study population by sex.

Variable	Total	Men		Women		χ2	*p*-value*
	*N*	*N*	%	*N*	%		
Age (years)							
19–30	312	150	15.3	162	15.2	8.646	0.071
31–50	844	426	43.4	418	39.1		
51–65	389	183	18.7	206	19.3		
66–75	426	195	19.9	231	21.6		
>75	78	27	2.8	51	4.8		
Place of residence							
Village	657	343	35.0	314	29.4	9.493	0.023
Town to 10 thousand	300	136	13.9	164	15.4		
Town 20–100 thousand	433	211	21.5	222	20.8		
City over 100 thousand	659	291	29.7	368	34.5		
Education							
Primary/junior high school/vocational	761	422	43.0	339	31.7	43.748	<0.001
Secondary (general or technical)	963	449	45.8	514	48.1		
Tertiary (bachelor’s degree, engineering studies, master’s degree)	325	110	11.2	215	20.1		
Financial situation**							
Good	496	291	29.7	205	19.2	31.719	<0.001
Average	1,371	616	62.8	755	70.7		
Poor	182	74	7.5	108	10.1		
Physical activity***							
Low	859	314	32.0	545	51.0	76.550	<0.001
Moderate	1,072	597	60.9	475	44.5		
High	118	70	7.1	48	4.5		
Current smoking							
No	1,427	641	65.3	786	73.6	16.478	<0.001
Yes	622	340	34.7	282	26.4		
Smoking in the past							
No	1,102	447	69.7	655	83.3	37.121	<0.001
Yes	325	194	30.3	131	16.7		
Current alcohol drinking							
Never	470	121	12.3	349	32.7	215.38	<0.001
1–3 times a month	765	326	33.2	439	41.1		
Once a week	465	283	28.8	182	17.0		
Several times a week	307	217	22.1	90	8.4		
Once daily	37	29	3.0	8	0.7		
Several times a day	5	5	0.5	0	0.0		

*Pearson’s chi-squared test. ** Financial situation: good: we are wealthy, we do not have to save even for larger expenses/we have enough money for all expenses, and we can save some; average: we have enough money for everyday expenses, but we cannot afford to spend more; poor: we have to deny ourselves a lot of things in order to have enough money to live, money is not enough even for the most urgent needs. *** Physical activity: Low: mostly sedentary, watching TV, reading newspapers/books, light house works, walking for 1–2 h/week; Moderate: walking, cycling, exercise, gardening or other light physical activity for 2–3 h/week; High: cycling, running, gardening and other sport/recreational activities that require physical activity for longer than 3 h/week.

The average energy value of men’s diets was significantly higher than in women (2547.42 ± 24.21 kcal vs. 1834.86 ± 19.87 kcal; *p* < 0.001). The average daily supply of dietary fiber was 17.83 ± 0.14 g/d in the study population, which constituted 78% of the demand for this ingredient. One-fifth of the respondents (20.53%) met the requirement for fiber, including significantly more men than women (27.05% vs. 14.3%, *p* < 0.001) (see Supplementary Table S1).

The diet of men was characterized by significantly higher fiber content than the diet of women (19.34 ± 0.20 g/d vs. 16.43 ± 0.19 g/d; *p* < 0.001). However, when standardizing the supply of fiber per 1,000 kcal of the diet, the diet of women turned out to contain significantly more fiber than the diet of men (9.31 ± 0.10 g/1000 kcal vs. 7.87 ± 0.08 g/1000 kcal; *p* < 0.001). The ratio of carbohydrates to dietary fiber was also significantly lower in the diet of women than in the diet of men (15.82: 1 ± 0.16 vs. 17.09: 1 ± 0.15; *p* < 0.001) (Table 3).

**Table 3:** Characteristics of the energy value of the diet and the average intake of dietary fiber and product groups that are fiber sources in the general population and by sex.

Variable	Total		Men		Women		*t*	*p*-value*
	M	SEM	M	SEM	M	SEM		
Total energy intake (kcal)	2176.92	17.53	2547.42	24.21	1834.86	19.87	23.10	<0.001
Total fiber (g)	17.83	0.14	19.34	0.20	16.43	0.19	10.52	<0.001
% of recommended intake	77.7	0.68	84.8	1.01	70.9	0.88	10.534	<0.001
Total fiber (g/1000 kcal)	8.62	0.07	7.87	0.08	9.31	0.10	−11.35	<0.001
Carbohydrate to fiber ratio	16.43:1	0.11	17.09:1	0.15	15.82:1	0.16	5.957	<0.001
Cereal (g) (total cereals)	189.79	1.83	220.82	2.55	161.15	2.28	17.749	<0.001
Cereal fiber (g)	7.67	0.08	8.48	0.11	6.90	0.11	10.347	<0.001
Refined bread (g)	121.28	1.94	160.74	2.88	84.85	2.03	21.930	<0.001
Refined bread fiber (g)	3.51	0.06	4.56	0.08	2.50	0.07	19.717	<0.001
Whole grain bread (g)	21.23	0.98	16.17	1.36	25.91	1.38	−4.889	<0.001
Whole grain bread fiber (g)	1.50	0.07	1.11	0.09	1.87	0.10	−5.236	<0.001
Vegetables (g)	250.26	3.43	267.66	5.09	234.20	4.57	4.766	<0.001
Vegetable fiber (g)	4.16	0.06	4.45	0.08	3.89	0.08	4.488	<0.001
Fruits (g)	173.02	3.48	145.64	4.78	198.30	4.90	−7.900	<0.001
Fruit fiber (g)	2.47	0.05	2.06	0.07	2.86	0.07	−8.215	<0.001
Legumes (g)	3.37	0.28	3.90	0.43	2.89	0.35	1.677	0.094
Legume fiber (g)	0.52	0.04	0.61	0.07	0.43	0.05	1.961	0.050
Nuts and seeds (g)	2.80	0.23	2.70	0.32	2.90	0.34	−0.108	0.914
Nuts fiber (g)	0.19	0.02	0.19	0.02	0.20	0.02	−0.015	0.988

M – mean, SEM – standard error of mean. * Student’s *t*-test.

The analysis of the intake of product groups being sources of dietary fiber showed a significantly higher intake of total cereal products, refined bread and vegetables in the group of men compared to the group of women (220.82 ± 2.55 g vs. 161.15 ± 2.28 g, 160.74 ± 2.88 g vs. 84.85 ± 2.03 g, 267.66 ± 5.09 g vs. 234.20 ± 4.57 g, *p* < 0.001). Conversely, women consumed significantly more whole grain bread and fruit compared to men (25.91 ± 1.38 g vs. 16.17 ± 1.36 g, 198.30 ± 4.90 g vs. 145.64 ± 4.78 g, respectively; *p* < 0.001) (Table 3).

**Figure 1:**
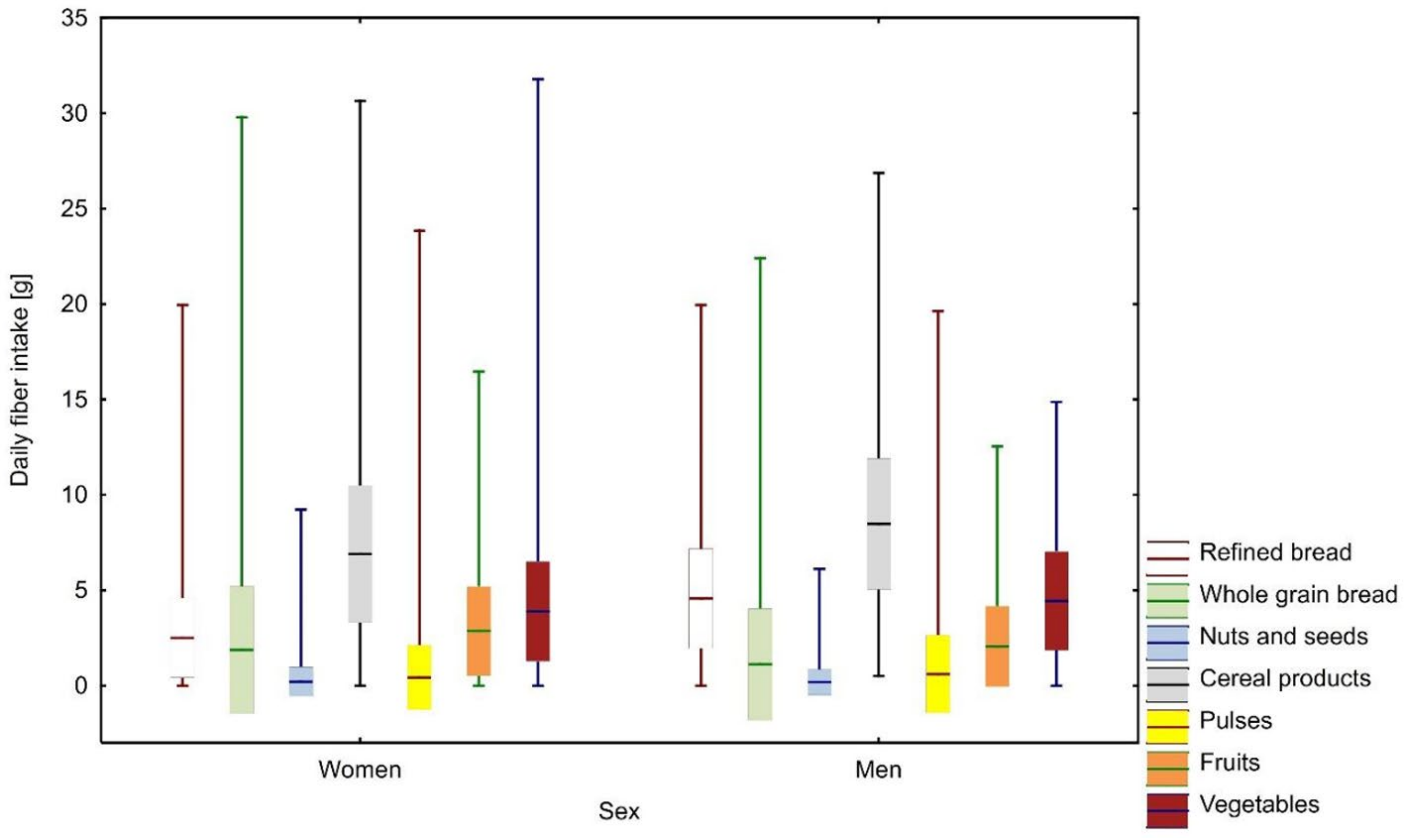
Average daily fiber intake by source among women and men: weighted means with standard deviation and measurement range.

The most fiber was obtained from total cereal products (44.1%) and vegetables (23.6%). Refined bread provided significantly more fiber in the diets of men than women (25.8% vs. 16.3%, *p* < 0.001). Conversely, fruit provided more fiber in the diets of women than men (17.2% vs. 10.2%, *p* < 0.001) (Figure 1).

A detailed analysis of dietary fiber supply in the diets of men depending on age showed several statistically significant differences. The diets of the youngest (19–30 years) and the oldest (>75 years) men were characterized by the highest average supply of fiber. Conversely, men aged 66–75 consumed significantly more fiber per 1,000 kcal of the diet than other age groups. The only group of men who fully met their fiber requirements were those aged >75 years. The youngest group of men was characterized by a significantly higher carbohydrate to fiber ratio compared to other age groups. As regards the amount of fiber consumed from specific product groups, significantly more fiber was obtained from total cereal products, nuts and seeds in men aged 19–30 compared to men from other age groups. The supply of dietary fiber from other product groups did not differ significantly depending on the age of the surveyed men (Table 4).

**Table 4:** The energy value of the diet, the percentage of energy from macronutrients and the intake of total dietary fiber and fiber from product groups that are its sources (men).

	Men					F	*p*-value*
	19–30	31–50	51–65	66–75	>75		
	M ± SD						
Total energy intake (kcal)	2967.52 ± 897.25	2595.01 ± 652.75	2449.64 ± 726.77	2203.23 ± 612.14	2478.54 ± 511.17	25.02	<0.001
Total fiber (g)	20.81 ± 6.92	19.18 ± 6.02	19.62 ± 6.22	18.18 ± 5.43	20.01 ± 5.07	3.93	0.004
% of recommended fiber intake	83.2 ± 27.68	76.7 ± 24.12	80.1 ± 26.64	90.9 ± 27.16	100.1 ± 25.35	12.00	<0.001
Total fiber (g/1000 kcal)	7.24 ± 2.09	7.62 ± 2.39	8.31 ± 2.53	8.52 ± 2.61	8.12 ± 1.4	8.42	<0.001
Carbohydrate to fiber ratio	18.36:1 ± 4.88	17.47:1 ± 4.48	15.91:1 ± 4.04	16.39:1 ± 4.73	16.74:1 ± 2.83	7.77	<0.001
Cereal (g) (total cereals)	255.94 ± 86.13	219.88 ± 70.31	214.17 ± 83.24	200.10 ± 70.92	232.02 ± 71.47	11.54	<0.001
Cereal fiber (g)	9.21 ± 3.01	8.5 ± 3.37	8.88 ± 4.07	7.75 ± 3.6	7.56 ± 3.64	4.74	0.001
Proportion of total daily fiber (%)	46.8 ± 14.46	45.9 ± 14.3	45.6 ± 13.36	43.9 ± 16.00	44.6 ± 17.14	0.90	0.466
Refined bread (g)	169.76 ± 94.43	163.42 ± 87.30	149.33 ± 94.65	157.70 ± 75.36	170.88 ± 76.89	1.35	0.251
Refined bread fiber (g)	4.64 ± 2.72	4.63 ± 2.51	4.59 ± 3.07	4.50 ± 2.37	4.95 ± 2.79	0.17	0.955
Proportion of total daily fiber (%)	24.3 ± 16.07	26.3 ± 15.48	24.6 ± 14.92	26.6 ± 14.21	25.7 ± 15.76	0.83	0.503
Whole grain bread (g)	12.70 ± 35.05	16.25 ± 40.95	24.11 ± 51.86	11.18 ± 33.69	13.06 ± 40.55	2.58	0.036
Whole grain bread fiber (g)	0.93 ± 2.56	1.15 ± 2.98	1.67 ± 3.61	0.79 ± 2.47	0.87 ± 2.58	2.28	0.059
Proportion of total daily fiber (%)	4.3 ± 11.20	4.9 ± 11.67	6.9 ± 13.94	3.9 ± 10.97	3.2 ± 8.71	1.77	0.132
Vegetables (g)	274.40 ± 156.07	267.49 ± 153.09	269.43 ± 153.24	257.79 ± 159.38	297.80 ± 158.49	0.43	0.791
Vegetable fiber (g)	4.58 ± 2.78	4.4 ± 2.49	4.46 ± 2.56	4.53 ± 2.85	4.76 ± 2.52	0.25	0.909
Proportion of total daily fiber (%)	21.8 ± 11.12	22.8 ± 10.24	23.6 ± 12.36	24.5 ± 12.80	27.8 ± 13.93	2.02	0.090
Fruits (g)	166.49 ± 150.49	132.77 ± 153.33	158.72 ± 145.31	145.22 ± 122.58	144.75 ± 107.03	1.91	0.107
Fruit fiber (g)	2.31 ± 2.21	1.9 ± 2.18	2.35 ± 2.25	2.12 ± 2.01	2.44 ± 2.77	2.02	0.090
Proportion of total daily fiber (%)	10.2 ± 8.76	9.0 ± 9.59	11.9 ± 10.88	11.8 ± 9.72	10.6 ± 8.43	4.07	0.003
Legumes (g)	2.15 ± 8.09	3.66 ± 13.61	4.37 ± 13.23	5.20 ± 14.21	5.56 ± 18.30	1.25	0.288
Legume fiber (g)	0.32 ± 1.24	0.6 ± 2.24	0.66 ± 2.04	0.74 ± 2.14	0.7 ± 2.41	0.97	0.424
Proportion of total daily fiber (%)	1.4 ± 5.49	2.4 ± 8.51	2.8 ± 8.53	3.8 ± 10.35	2.9 ± 9.53	1.62	0.168
Nuts and seeds (g)	5.40 ± 15.07	2.81 ± 9.59	2.22 ± 8.23	0.76 ± 4.51	2.42 ± 7.40	4.70	0.001
Nuts fiber (g)	0.39 ± 1.13	0.18 ± 0.57	0.15 ± 0.52	0.09 ± 0.46	0.13 ± 0.58	4.93	0.001
Proportion of total daily fiber (%)	1.5 ± 3.95	0.8 ± 2.58	0.6 ± 2.08	0.4 ± 1.94	0.8 ± 3.23	3.86	0.004

*one way ANOVA. M – mean, SD – standard deviation.

No statistically significant differences were shown as regards the supply of total dietary fiber with the diets of women depending on age. However, after converting the amount of fiber consumed per 1,000 kcal of the diet, its consumption appeared to be significantly higher in women aged 51–65 years. In addition, this age group of women was characterized by the lowest ratio of carbohydrates to fiber. The analysis of meeting the requirements concerning dietary fiber consumption showed that the reference value of sufficient intake was met to the highest extent by women aged 66–75. An in-depth analysis of dietary fiber intake from different product groups depending on age revealed significant differences only in case of total cereal products and refined bread. Women aged 51–65 consumed significantly more fiber with total cereal products, and women aged >75 years with refined bread (Table 5).

**Table 5:** The energy value of the diet, the percentage of energy from macronutrients and the intake of total dietary fiber and fiber from product groups that are its sources (women).

	Women					F	*p*-value*
	19–30	31–50	51–65	66–75	>75		
	M ± SD						
Total energy intake (kcal)	1862.83 ± 689.86	1860.68 ± 630.42	1807.47 ± 620.09	1799.34 ± 597.56	1783.44 ± 587	0.57	0.685
Total fiber (g)	16.4 ± 7.29	16.3 ± 6.17	16.94 ± 5.54	16.37 ± 5.52	15.42 ± 6.22	0.68	0.604
% of recommended fiber intake	65.6 ± 29.16	65.3 ± 24.67	68.7 ± 22.14	81.8 ± 27.62	77.1 ± 31.10	15.91	0.001
Total fiber (g/1000 kcal)	9.13 ± 3.5	9.05 ± 3.15	9.93 ± 3.34	9.45 ± 2.74	8.71 ± 2.27	3.26	0.011
Carbohydrate to fiber ratio	16.68:1 ± 5.84	15.93:1 ± 5.09	14.95:1 ± 4.34	15.68:1 ± 4.60	16.52:1 ± 4.50	2.98	0.018
Cereal (g) (total cereals)	162.67 ± 74.60	157.47 ± 72.20	163.17 ± 68.55	166.95 ± 76.74	153.24 ± 55.40	0.76	0.548
Cereal fiber (g)	6.95 ± 3.66	6.74 ± 3.71	7.2 ± 3.55	6.92 ± 3.92	6.36 ± 3.25	0.81	0.520
Proportion of total daily fiber (%)	43.6 ± 14.25	41.9 ± 15.44	42.5 ± 14.48	41.7 ± 14.32	42.7 ± 13.47	0.48	0.748
Refined bread (g)	72.45 ± 59.73	78.63 ± 64.01	89.47 ± 64.43	97.92 ± 64.97	105.87 ± 65.36	5.88	<0.001
Refined bread fiber (g)	2.15 ± 1.96	2.29 ± 2.11	2.62 ± 2.01	2.88 ± 2.07	3.15 ± 2.25	5.00	0.001
Proportion of total daily fiber (%)	15.4 ± 13.83	14.9 ± 12.24	16.1 ± 12.00	18.7 ± 12.99	21.0 ± 13.12	4.68	0.001
Whole grain bread (g)	24.30 ± 44.37	27.14 ± 43.19	30.76 ± 46.84	20.92 ± 41.90	19.94 ± 37.98	1.60	0.172
Whole grain bread fiber (g)	1.79 ± 3.28	1.97 ± 3.38	2.31 ± 3.70	1.49 ± 3.02	1.41 ± 2.83	1.79	0.130
Proportion of total daily fiber (%)	9.2 ± 14.30	10.8 ± 15.98	12.4 ± 17.95	8.0 ± 14.26	8.9 ± 15.68	2.38	0.050
Vegetables (g)	225.65 ± 144.31	237.01 ± 159.85	242.25 ± 126.33	235.01 ± 135.47	193.32 ± 104.69	1.13	0.340
Vegetable fiber (g)	3.72 ± 2.45	3.93 ± 3.07	3.92 ± 2.11	4.16 ± 2.54	3.46 ± 2.96	1.06	0.377
Proportion of total daily fiber (%)	23.1 ± 13.06	24.1 ± 13.18	23.7 ± 10.93	25.5 ± 12.50	21.9 ± 10.97	1.29	0.272
Fruits (g)	187.23 ± 177.36	194.25 ± 150.90	203.49 ± 147.67	204.95 ± 150.59	221.60 ± 166.72	0.64	0.636
Fruit fiber (g)	2.83 ± 2.87	2.79 ± 2.31	2.92 ± 2.19	2.84 ± 2.09	3.25 ± 2.93	0.48	0.751
Proportion of total daily fiber (%)	15.7 ± 12.46	16.8 ± 11.54	17.6 ± 12.13	18.2 ± 12.24	20.2 ± 13.82	1.72	0.143
Legumes (g)	2.85 ± 11.01	2.93 ± 10.14	2.99 ± 10.97	3.09 ± 13.89	1.09 ± 4.88	0.28	0.890
Legume fiber (g)	0.38 ± 1.53	0.4 ± 1.45	0.46 ± 1.69	0.57 ± 2.38	0.13 ± 0.68	0.82	0.514
Proportion of total daily fiber (%)	1.8 ± 6.69	2.0 ± 7.00	2.4 ± 8.36	2.0 ± 7.26	1.0 ± 4.71	0.40	0.812
Nuts and seeds (g)	3.11 ± 15.44	3.48 ± 10.79	2.90 ± 8.88	1.48 ± 6.11	3.22 ± 14.48	1.20	0.309
Nuts fiber (g)	0.21 ± 1.08	0.22 ± 0.72	0.19 ± 0.63	0.12 ± 0.52	0.22 ± 1.05	0.67	0.615
Proportion of total daily fiber (%)	1.0 ± 3.97	1.3 ± 3.64	0.9 ± 2.74	0.7 ± 2.50	1.5 ± 5.28	1.27	0.282

*one way ANOVA. M – mean, SD – standard deviation.

## 4 Discussion

The research revealed an insufficient intake of fiber in the majority of Polish adults. The average fiber supply was only 17.83 g/d. The diet of men (M) was characterized by significantly higher fiber content than the diet of women (W) (M: 19.34 g/d vs. W: 16.43 g/d), which may be explained by the higher average energy value of the men’s diet. The analysis of fiber supply depending on the age of the respondents showed that the diets of the youngest (19–30 years) and oldest (>75 years) men contained the most fiber. The diets of women aged 51–65 were characterized by the highest fiber density. Only every fifth (20.03%) respondent met the requirement for fiber at the level of the sufficient reference intake. The only group of men who fully met their fiber requirements were those aged >75 years. The obtained results are difficult to compare directly to the reports of other researchers. It is mainly due to differences in the methodology of collecting data on diet and a less detailed method of data analysis. Nevertheless, our results stay in line with the results of studies conducted in European countries (21) and the United States (31), which indicates that the problem of low fiber in the diet affects numerous populations and is associated with common/global changes in lifestyle, including the diet (32, 33). Comparing the results of our study to the results of previous Polish epidemiological studies in which fiber supply was calculated on the basis of daily intake, insufficient fiber intake was observed in the general population and was lower by 1.54 g/d in men. Moreover, the percentage of men meeting the fiber requirements was lower by 1.75% (22). It should be emphasized that the discussed studies did not invoke the reference values of sufficient fiber supply which determine the amount of the ingredient covering the needs of healthy and properly nourished people (12). These reference values primarily take account of the role of fiber in the proper functioning of the gastrointestinal tract. However, researchers agreed that fiber intake that was higher than sufficient strongly corresponded to a reduction in the risk of certain chronic diseases, including cardiovascular diseases, stroke, type 2 diabetes, colorectal cancer and diverticular disease. Moreover, the risk of death due to any cause was 15–16% lower in individuals who consumed large amounts of fiber compared to those who consumed lower amounts. It was confirmed that the intake of 14 g of fiber per 1,000 kcal of food protected against cardiovascular disease (34). Analyses indicated that a daily fiber intake of 25–29 g was adequate, but an intake of over 30 g per day would be even more beneficial (35). Reynolds et al. noted that a fiber intake of over 35 g per day appeared to be more effective than a lower intake in reducing the risk of cardiovascular disease, type 2 diabetes, and colorectal and breast cancer (36). In addition, the results of some studies indicated that the supply of about 50 g of fiber per day was correlated with a very low risk of colon cancer (37). Therefore, an increase in dietary fiber supply might be an important preventive factor since chronic non-communicable diseases constitute the main causes of death and lost years of healthy life in the Polish population (38).

In this paper, the supply of dietary fiber was also characterized by presenting the ratio of the total carbohydrate content to the fiber content in the diet. The ratio is a good measure of the quality of the diet (39, 40). According to the American Heart Association, this ratio should ideally be ≤10:1, which would provide 30 g of fiber in case of a 2000 kcal diet (41). It was demonstrated that a low ratio of carbohydrates to dietary fiber was associated with lower fat mass (42). The obtained results indicated that the Polish diet deviated far from those recommendations. In the general population, the ratio was 16.43:1. It was significantly higher in men than in women (M: 17.09:1 vs. W: 15.82:1), and the highest value was obtained in the group of the youngest men (18.66:1). To compare, a study conducted in Australia to analyze the ratio of carbohydrates to fiber in individual products revealed that the optimal ratio of 10:1 was achieved by half of the products consumed by adults (43).

The choice of products, cereal ones in particular, is of great importance in achieving the optimal amount of fiber and the ratio of carbohydrates to fiber in the diet. Our study showed that 220 g/d and 161 g/d of cereal products were consumed by men and women, respectively. It means a decrease compared to the consumption recorded in the WOBASZ study conducted in 2003–2005 (by 80 g/d and 119 g/d, respectively) (44). The decreased consumption of cereal products in Poland in the years 2010–2018 by more than 30% was also demonstrated in the Report of the National Institute of Public Health – National Institute of Hygiene (NIPH NIH), based on the results of household budget surveys conducted by the Statistics Poland (45).

Despite the observed reduction in the consumption of cereal products in Poland, they still remain the main source of fiber, providing 44.1% of the amount consumed. Similar amounts of fiber (48–49%) from cereal products are provided with diets in Ireland, the Netherlands and Sweden, and slightly lower (32–33%) in the USA and Spain (21). Evidence obtained from observational studies indicated that a higher intake of fiber from cereal sources (mainly whole grains) provided a greater degree of protection against the development of type 2 diabetes, which was probably due to its interaction with the intestinal microbiota or insulin-sensitizing components found in whole grains (35). Regrettably, our study showed that only 7.5% of the total amount of fiber was obtained from whole grain bread. Women consumed significantly more whole grain bread compared to men (W: 25.91 ± 1.38 g/d vs. M: 16.17 ± 1.36 g/d, *p* < 0.001), and that source provided them with over 1.5 times more fiber. In the studied population, the highest share of whole grain bread fiber in daily consumption was noted in the group of women aged 51–65 (12.4%).

The obtained results indicate that whole grain bread, which is a rich source of fiber, was consumed in small quantities, which largely contributed to the low level of fiber intake in the diet. The preference for refined bread leads to a situation in which this type of bread becomes the main source of fiber in the diet. If refined bread was replaced by whole grain one, the total supply of fiber could increase significantly. The average consumption of refined bread in the general population was about 120 g per day and provided 3.5 g of fiber. If refined bread was replaced by whole grain bread containing about 7 g of fiber per 100 g of product, the daily fiber intake could increase by about 5 g per day, which would significantly bring its total intake closer to the recommended values.

Vegetables and fruits followed cereal products in the Polish diet. They provided 23.6 and 13.8% of total fiber, respectively. This stays in line with the results of Stephen et al., who indicated that vegetables provided 12–21%, and fruits provided 8–23% of fiber intake in the diets of Europeans (21). The present study showed that the average total intake of fruit and vegetables in the whole group was 423.28 g/d and was close to the desired value, i.e., at least 400 g according to the WHO (46). A significantly higher intake of vegetables was noted in the group of men compared to the group of women (M: 267.66 ± 5.09 g/d vs. W: 234.20 ± 4.57 g/d; *p* < 0.001). Women consumed significantly more fruits compared to men (W: 198.30 ± 4.90 g/d vs. M: 145.64 ± 4.78 g/d, *p* < 0.001). The obtained results are comparable to the results of the 2003–2005 WOBASZ study (44) and slightly higher than those presented by the NIPH NIH, where the total consumption of fruits and vegetables was 313–315 g/d in 2017–2018 (45).

Although the participants of the present study consumed a total of 423 g/d of fruits and vegetables, this result cannot be considered satisfactory. Consuming additional 100 g of vegetables (containing about 2–4 g of fiber) and 100 g of fruit (containing about 2–3 g of fiber) could significantly improve daily fiber intake. Fruits and vegetables are not only a source of dietary fiber, but also vitamins, minerals and numerous beneficial phytonutrients, including plant sterols, flavonoids and other antioxidants. Lower consumption of fruits and vegetables is associated with an increased risk of non-communicable diseases. It was estimated that in 2017, 3.9 million deaths worldwide could be attributed to the insufficient consumption of fruit and vegetables (46, 47). It was shown that each additional 200 g of fruits and vegetables per day was associated with a 16% reduction in the risk of stroke, 8% reduction in the risk of coronary heart disease and cardiovascular disease, 3% lower risk of cancer, as well as a 10% lower risk of premature death. The consumption of up to 800 g of fruits and vegetables per day was associated with a reduced risk of cardiovascular disease, and up to 600 g – with a decreased risk of developing cancer (48).

The consumption of other product groups analyzed in our study, i.e., legume seeds, nuts and seeds were dramatically low. These products certainly cannot be considered as significant sources of dietary fiber in the Polish diet. The consumption of legume seeds was at the level of 3.37 ± 0.28 g/d, and in case of nuts and seeds it was 2.8 ± 0.23 g/d, without sex-related differences. The obtained results were confirmed in the literature. According to the Global Dietary Database, the consumption of legumes in Poland was 3.0 g/d, which placed it among countries with the lowest overall consumption of this group of products (Norway – 1.2 g/d, Switzerland – 2.9 g/d, Argentina – 3.7 g/d and Malta – 4.0 g/d). In contrast, in countries with the highest consumption of legume seeds, their consumption was recorded at a level above 100 g/d (Afghanistan – 122.7 g/d, Vietnam – 104.7 g/d) (49). Legumes, nuts and seeds of other plants are characterized by exceptionally high nutritional value, and their proper share in the diet was associated with confirmed health benefits (50, 51). 100 g of nuts contain between 6 and 13 g of fiber, so consuming the recommended daily portion of 30 g of nuts could increase fiber intake by an additional 1.8 to 3.9 g of fiber per day. The suggested target intake of legume seeds is set at 50 g/d (46), and unsalted nuts at 30 g/d (52). Low interest in legume dishes in Poland is associated with the lack of habit of eating them, discomfort in the gastrointestinal tract after consumption, or long preparation time. Szczebyło et al. demonstrated that the improvement in the frequency of their consumption in Poland could be influenced by: the knowledge of new recipes with their use, gaining the ability to prepare dishes, no discomfort after eating, lower prices of products offered on the market, gaining knowledge about the nutritional value of legumes and a wider range of dishes in restaurants (53). Achieving these goals requires the implementation of widely available culinary education to improve knowledge of these important health products, the ability to prepare them is a proper way, and the resultant modification of shopping habits, and more frequent inclusion of legumes in the diet (49).

Despite numerous health benefits of dietary fiber, only a low percentage of people follow a diet in which products being good fiber sources are found in optimal amounts. When looking for the reasons for the low supply of dietary fiber in the Polish diet, it is necessary to pay attention to the multifactorial dimension of this problem. Undoubtedly, the global rising trend in the popularity of highly-processed products (3, 54), the consumption of which is inversely correlated with the consumption of dietary fiber, is one of underlying factors (55). Another factor explaining the low fiber content of diets may be related to the popularity of low-carbohydrate diets, which assume a reduction in the amount of carbohydrates consumed. This is achieved by reducing the consumption of sugar-rich processed products, but also cereal products (bread, pasta, groats, rice), some fruits and vegetables, as well as pulses, which are a source of fiber (56). It is a common myth among consumers that carbohydrates promote weight gain, which is due to misunderstanding the difference between complex and simple carbohydrates. Initiatives to update the guidelines for carbohydrate quality assessment and to identify “high quality” carbohydrate sources as derived from whole grains, legumes, fruits and vegetables seem to be of particular importance (57).

Optimizing fiber intake at the population level requires a multifaceted approach that takes account of various factors affecting eating habits and involves the use of innovative strategies. In addition to effective educational programs on the health benefits of a high-fiber diet, it is necessary to develop strategies that will encourage the selection of high-fiber products in an attractive way for the modern consumer.

The most appropriate way to increase the amount of fiber in the diet is to choose whole grains instead of refined cereal products. The key to achieving this goal is to increase the availability of high-fiber foods in stores, such as whole grain bread and pasta, at the same time reducing the presence of highly processed products. It is also important to clearly label healthier dietary options, which will make it easier for consumers to make more beneficial choices. In addition, it is important to reduce the economic barrier associated with the higher costs of whole grains compared to processed products. Willett et al. suggested that the target daily intake of whole grains should be 232 g (58). However, this would require a radical change in dietary patterns and is unlikely due to numerous barriers (59). In this situation, it may be helpful to implement an approach focused on personalized dietary advice, adapting nutritional suggestions to individual habits, leading to greater adherence to fiber-related recommendations (60). Incorporating fiber-enriched foods into the daily diet, without compromising taste or consumer acceptance may also be an effective strategy to significantly increase fiber intake. A study by Brandl et al. revealed that offering a variety of fiber-enriched foods increased daily fiber intake by 10–12 grams in middle-aged individuals with and without cardiometabolic risk (61). Moreover, an increase in fiber intake may be achieved by introducing new technologies and promoting sustainable food production methods. Enriching 3D printed apricot gel snacks with fiber derived from orange waste may be an example of such a technology. Citrus waste, such as orange peels, is rich in nutrients, including fiber and phenolic compounds. Their reuse in food production, for example by adding them to novel foods, may increase the intake of fiber and bioactive compounds while promoting a more sustainable use of resources (62). Aiming at increasing the consumption of legumes may be another strategy. It would be realistic to implement the recommendations to consume half of cereal products in the form of whole grains and to consume one portion of legumes per day (63). A portion of 100 g of legumes provides about 7 g of fiber. Legumes may be included in the diet not only in the form of seeds, but also processed, in the form of flour, pasta or products with their addition. The consumption of fruits and vegetables (fresh and frozen) should also be constantly promoted as part of each meal throughout the year, in accordance with current recommendations.

All these strategies should be supported by government policies promoting the production and promotion of fiber-rich foods through subsidies, educational campaigns, and legislative changes that will facilitate access to healthy foods. The implementation of such strategies may significantly contribute to increasing the intake of fiber in the population, leading to better health outcomes and supporting sustainable eating habits. Thus, the research indicates that increasing population fiber intake requires a differentiated approach that includes consumer education, collaboration with the food industry, and government support. Examples such as replacing refined bread by whole grain one, promoting the consumption of legumes and fruit and vegetables, as well as the use of innovative technologies and promotional strategies may contribute to increasing fiber intake and improving public health.

### 4.1 Strengths and limitations

This study boasts several robust attributes that enhance its scientific merit and reliability. First, it employs a large, nationally representative sample, which improves the generalizability of the findings to the adult population of Poland. The inclusion of 4,000 participants from diverse demographic backgrounds ensures a comprehensive analysis across different age, sex, and socioeconomic groups, thereby enhancing the external validity of the study. Secondly, the methodological rigor of this research is evident in its use of a validated 24-h dietary recall and the Computer-Assisted Personal Interview (CAPI) techniques. These methods are well-established in nutritional epidemiology for their accuracy and reliability in dietary assessment. The study also benefits from the dual-recall approach, where dietary intake was assessed twice per participant, thus reducing the recall bias commonly associated with single dietary assessments. Furthermore, the statistical analysis was meticulously conducted with weighted analysis to account for the stratified sample design, ensuring that the findings were reflective of the national population structure. This level of detail in statistical handling allows for a more accurate interpretation of dietary patterns and fiber intake across various subgroups.

The study faces certain limitations that merit consideration. One of the primary limitations is related to its cross-sectional design, which, while effective for assessing dietary patterns at a specific point in time, does not allow for causal inferences between dietary fiber intake and health outcomes. Longitudinal studies would be necessary to establish causality and observe changes in dietary habits over time. Additionally, the reliance on self-reported data through 24-h dietary recalls may introduce reporting bias, typically underreporting or overreporting food intake, which might affect the accuracy of the dietary assessment. Although the use of a validated tool and repeated measures helps mitigate this issue, it cannot completely eliminate the inherent biases associated with self-reported data. Another limitation is linked to the potential for residual confounding by factors not adequately controlled or assessed in the study, such as detailed socioeconomic status, psychological factors, and other lifestyle variables like sleep patterns and sedentary behaviors, which might influence dietary habits and fiber intake.

While this study provides valuable insights into the dietary fiber intake among Polish adults and identifies key demographic disparities, the results must be interpreted within the context of its design limitations. Future research would benefit from longitudinal designs and a more extensive assessment of confounding factors to deepen the understanding of the dynamics and implications of fiber intake in the diet.

## 5 Conclusion

The study revealed that the average daily intake of fiber among the adult population in Poland was below the recommended levels in virtually all age groups of men and women. The main source of this component is cereal products, although the intake of whole grain cereal products is low. Vegetables are also important sources of fiber, but pulses and nuts are of marginal importance.

Our study reflects global observations that fiber intake is generally inadequate in different populations. Addressing this widespread dietary deficiency necessitates enhanced public health education focusing on the benefits and sources of dietary fiber and methods for its incorporation into daily meals. Governments and health organizations should consider policies that encourage the production and consumption of high-fiber foods such as whole grains, fruits, and vegetables. Subsidies for whole grains and promotional campaigns highlighting their health benefits could significantly improve public dietary habits. Special interventions might also be required to target specific demographics such as younger adults and women to normalize higher fiber consumption relative to their energy intake.

Given the universal challenge posed by inadequate fiber intake, there is a compelling case for global health campaigns and food policies aimed at increasing fiber consumption. While the specific types of fiber-rich foods might vary across cultures, the fundamental strategies, i.e., emphasizing whole grains, fruits, and vegetables, may be applied universally, albeit with cultural adaptations to enhance effectiveness.

## Data Availability

The raw data supporting the conclusions of this article will be made available by the authors, without undue reservation.
