# Supplementary material for: Assessing the impact of dietary choices on fiber deficiency: insights from the 2017–2020 Polish national adult nutrition survey
Source: Front Nutr. 2024 Sep 13;11:1433406. doi: 10.3389/fnut.2024.1433406 (PMC11427414; doi:10.3389/fnut.2024.1433406)
Supplement: Supplementary file 1 [file Table_1.DOCX]

Supplementary Material

# Supplementary Table

SUPPLEMENTARY TABLE 1 Percentage of individuals meeting the reference ranges for dietary fiber intake.

| Are the reference ranges met? | Women | Men | Total (no/yes) |
| --- | --- | --- | --- |
| no | 85.70% | 72.95% | 79.47% |
| yes | 14.30% | 27.05% | 20.53% |
| Total (women/men) | 51.13% | 48.87% | 100.00% |

|  | Chi squared | p |
| --- | --- | --- |
| Pearson's Chi^2 | 49.71385 | < 0.001 |

OR = 2.22; 95%CI [1.77; 2.78]
